# Coronary Computed Tomography Angiography (CTA) Findings in COVID-19

**DOI:** 10.3390/jcdd11100325

**Published:** 2024-10-14

**Authors:** Pietro G. Lacaita, Anna Luger, Fabian Plank, Fabian Barbieri, Christoph Beyer, Theresa Thurner, Yannick Scharll, Johannes Deeg, Gerlig Widmann, Gudrun M. Feuchtner

**Affiliations:** 1 Department Radiology, Innsbruck Medical University, 6020 Innsbruck, Austria; placaita@gmail.com (P.G.L.); christoph.beyer@i-med.ac.at (C.B.); yannick.scharll@i-med.ac.at (Y.S.); johannes.deeg@i-med.ac.at (J.D.); gerlig.widmann@tirol-kliniken.at (G.W.); 2 Department Internal Medicine, Tyrol Clinicum Hall, 6060 Hall, Austria; fabian.plank@tirol-kliniken.at; 3 Department of Cardiology, Charité—Universitätsmedizin Berlin, Corporate Member of Freie Universität Berlin and Humboldt-Universität zu Berlin, Hindenburgdamm 30, 12203 Berlin, Germany; fabian.barbieri@dhzc-charite.de; 4 Gesundheitszentrum Lanserhof, 6072 Lans, Austria; theresa.thurner@gmail.com

**Keywords:** coronary computed tomography angiography (CTA), COVID-19, SARS-CoV-2, vasculitis, coronary artery disease, chest pain, myocardial injury, inflammation

## Abstract

(1) Background: The novel SARS-CoV-2 virus infects the endothelium. Vasculitis may lead to specific coronary artery wall lesions. Coronary computed tomography angiography (CTA) imaging findings have not been systematically reported. The aim of this study was to describe a case series using CTA. (2) Methods: Patients with recent RT-PCR confirmed SARS-CoV-2 infection referred for coronary CTA for clinical indications (e.g., chest pain, troponin+, and ECG abnormalities) were included. Coronary CTA findings, such as atypical coronary lesions suggestive of vasculitis, perivascular inflammation measured by using pericoronary fat attenuation (PCAT) index, coronary artery disease, and extracoronary findings were collected. (3) Results: Results for 12 patients (54.8 ± 22 years; four females) with SARS-CoV-2 infection within 60 days (four acute care and eight stable patients) are reported. Time to positive RT-PCR was a mean of 15.1 days (range, 0–51). In four acute patients with signs of myocardial injury, plaque rupture (n = 1), hyperenhancing myocardium/MINOCA (n = 1), MINOCA (n = 1), and pericarditis with acute heart failure (LVEF 20%) (n = 1) were found. All (100%) had pericardial effusion and signs of perivascular inflammation. Among eight stable patients, pericardial effusion or perivascular inflammation were found in only two (25%). Coronary artery disease was ruled out in five (62.5%) (4) Conclusions: Coronary CTA is a useful imaging modality in the diagnostic work up of patients with COVID-19 infection, and is able to describe coronary and other cardiac abnormalities.

## 1. Introduction

The coronavirus disease (COVID-19) pandemic has reshaped the world, and created new challenges for healthcare systems globally. COVID-19 is caused by novel severe acute respiratory syndrome coronavirus 2 (SARS-CoV-2) and affects various structures of the cardiovascular system, within the heart and blood vessels. By entering via angiotensin converting enzyme-2 (ACE-2) receptors, both the myocardium and the endothelium can be infected. Patients may develop an acute COVID-19 cardiovascular syndrome, mimicking myocardial infarction, but can also present with myocarditis. Troponin elevations are observed, which are mostly mild, but a differential diagnosis to other diseases such as coronary heart disease, is of great clinical importance. The cause of this myocardial injury in COVID-19 is possibly related to myocarditis, microvascular injury, systemic cytokine-mediated injury, and/or stress-related cardiomyopathy. Endothelitis and microthrombi [1] may lead to specific wall lesions with signs of vasculitis or myocardial infarction in non-obstructive coronary arteries (MINOCA). The destabilization of atherosclerotic plaque due to local and/or systemic inflammation, as recently reported by Ciliberti et al. [2], represents another potential mechanism of MINOCA in COVID-19 patients. Coronary computed tomography angiography (CTA) allows for an accurate evaluation of coronary arteries including vessel walls and lumen, with a high temporal and spatial resolution of 0.3–0.4 mm^3^. Coronary stenosis and coronary artery plaque can be visualized and quantified. Coronary CTA also allows for an evaluation of specific signs of vasculitis, such as atypical wall lesions (for example, wall thickening, focal aneurysm, or ectatic segments, and signs of perivascular inflammation). Pericoronary fat attenuation (PCAT) [3], is a novel quantitative marker for perivascular inflammation. An increased PCAT indicates perivascular inflammation, either due to systemic vasculitis, or to a single focal lesion. In addition, extracoronary findings such as pericardial effusion can be detected by coronary CTA indicators for ongoing cardiac inflammation.

Not only during acute COVID-19 infection, but also after the acute phase, the cardiovascular system may remain involved and cause symptoms known as “long COVID”. A large UK cohort study with 428,650 participants found that, after COVID-19, patients have adverse long-term cardiovascular outcomes, with a net increase in cardiovascular disease (CVD) incidence (including pulmonary embolism, atrial arrhythmias, and venous thrombosis) after 4 weeks [4], and an increase in the incidence of diabetes for at least 12 weeks, which was then found to be declining. Overall, the diagnosis of diabetes increased by 81%. Diabetes is a known main driving factor for the accelerated formation of coronary atherosclerosis. As well, endothelial dysfunction has been reported in long COVID patients. Most recently, Raafs et al. [5] reported long-term cardiac sequelae after 6 months by cardiac magnetic resonance (CMR) in 42% of 52 intensive care unit (ICU) survivors. Abnormal CMR findings were mostly non-ischemic late gadolinium enhancement consistent with perimyocarditis, while mildly reduced left ventricular ejection fraction (EF) was less common. Of note, one third of the patients had troponin elevations during severe COVID-19 infection.

Chest pain is common during and after COVID-19. Coronary CTA is the modality of choice for ruling out or detecting underlying coronary artery disease (CAD) [2]. Recognizing specific CTA imaging findings caused by SARS-CoV-2 infection is of importance for both radiologists and clinicians, in order to relate cardiac symptoms such as chest pain complaints, troponin elevations, and ECG abnormalities to either COVID-19 or underlying CAD, thromboembolic events, or perimyocarditis.

Coronary computed tomography angiography (CTA) findings during COVID-19 have not been reported in a case series. Currently, only a few reports using coronary CTA in COVID-19 exist in the literature [6]. One case showed classic MINOCA resembling an atypical Takotsubo cardiomyopathy, and presented with chest pain and mild troponin elevation, resembling non-ST elevation myocardial infarct (NSTEMI). The differential diagnosis of MINOCA and myocarditis is challenging in those patients, and multimodality imaging (with both cardiac magnetic resonance imaging (CMR) and computed tomography angiography (CTA)) is an appropriate strategy for clinical work-up.

Therefore, we aim to describe coronary CTA imaging findings in a larger case series with precise serial imaging illustrations (pictorial essay).

## 2. Materials and Methods

We enrolled patients with recent SARS-CoV-2 infection who were referred for ECG-gated coronary CTA between March 2020 and November 2021 for clinical indications such as acute or stable chest pain [7] and suspected coronary artery disease based on pre-test probability and clinical likelihood. In acute patients with troponin elevation and NSTEMI-ACS, CTA was used as an alternative modality to invasive catheterization (ICA) during the early COVID-19 pandemic (2020–2021) and restricted resources. A 128-slice dual-source computed tomography (CT) (Definition FLASH or DRIVE, Siemens Healthineers, Erlangen, Germany) was used, equipped with a detector collimation of 2 × 64 × 0.6 mm and a gantry rotation time of 0.28 s. The CT image acquisition was triggered into the arterial phase by placing a region of interest (ROI) into the ascending aorta. We applied bolus tracking during contrast agent injection. The threshold was set at 100 HU. An iodinated intravenous contrast agent (Iopromide, Ultravist 370™, Bayer Healthcare, Berlin, Germany) was injected. Prospective electrocardiogram (ECG)-triggering in sequential scan mode was applied in patients with regular heart rates <65 beats per minute (bpm) (70% of RR interval) and retrospective ECG-gating was utilized in those with >65 bpm and irregular rhythm. Axial thin slice images were reconstructed at 0.75 mm slice width, and with overlapping slices with an increment of 0.4. Axial images were transferred to an advanced cardiovascular three-dimensional (3D)-postprocessing software (SyngoVIA VB80D, Siemens Healthineers, Erlangen, Germany).

Coronary arteries were evaluated using curved multiplanar reformations (MPR) for typical atherosclerotic plaque, stenosis > 50%, high-risk plaque (HRP) features, and atypical lesions including signs of vasculitis (focal or diffuse ectasia, wall thickening, and perivascular inflammation). High-risk plaques (HRP) were defined according to standardized criteria (low attenuation plaque < 30 Hounsfield Units (HU) and <60 HU, positive remodeling, spotty calcification, and Napkin-Ring Sign) [8]. The pericoronary fat attenuation (PCAT) index was quantified (left coronary artery (LCA), right coronary artery (RCA), and lesion-specific) and defined as positive for perivascular edema indicating inflammation if below −70 HU [3]. Extracoronary findings were recorded (pericardial effusion and others such as lung involvement) according to clinical standardized reporting.

## 3. Results

Of 19 patients who underwent CTA, 12 patients (four acute care patients and eight patients with stable chest pain) (age, 54.8 ± 22 years; four females) with SARS-CoV-2 infection within 60 days were included (Table 1). The time interval from first diagnosis (SARS-CoV-2 positive RT-PCR) to CTA was a mean of 15.1 days (range, 0–51 days).

**Figure 1:**
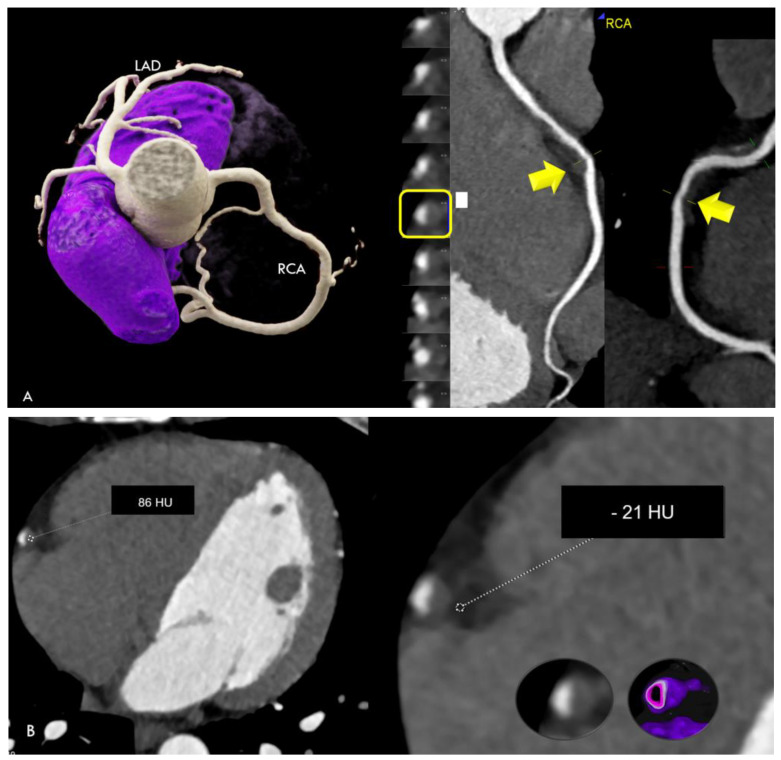

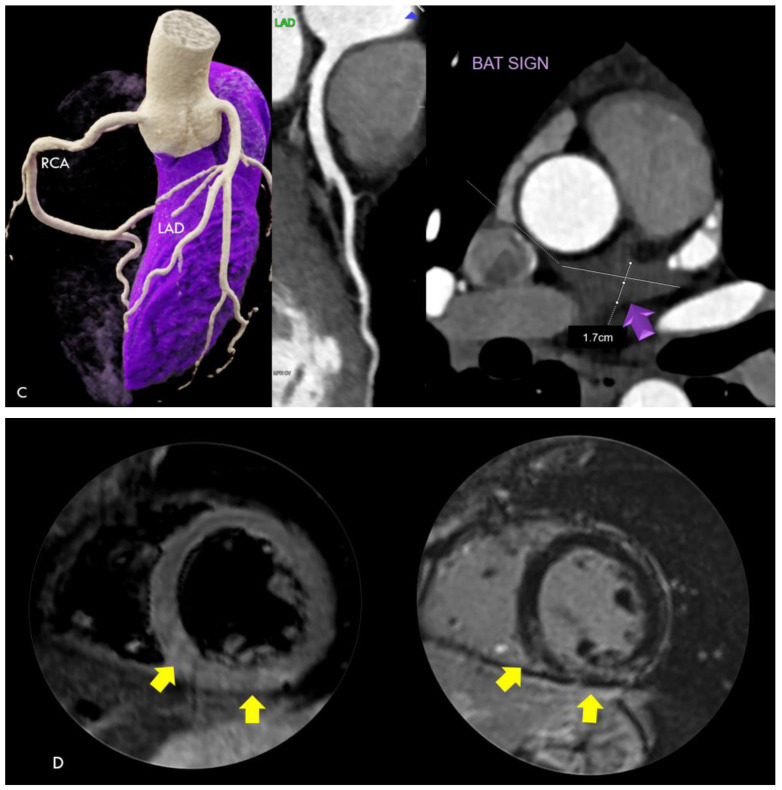
(**A**) Coronary CTA findings: Non-calcified hypodense lesion with low CT attenuation and 40% area stenosis in the mid right coronary artery (RCA) (arrow). (**left**)*:* Three-dimensional Volume Rendering Technique (3D-VRT). (**right**): Curved multiplanar reformations (cMPR). (**B**) The non-calcified lesion had low CT attenuation (86 HU) (**left**). Lesion-specific PCAT was positive (**right**) for perivascular edema (−21 HU), measured by a region of interest (ROI). (**C**) Left anterior descending (LAD), right coronary artery (RCA), and all other coronary arteries were normal ((**left**)*:* 3D-VRT and (**mid**): cMPR). Calcium score was zero. Prominent pericardial fluid (“bat sign”) within the superior epicardial recess *(***right**). (**D**) Cardiac MRI showed myocardial edema (**left**) on turbo inversion recovery magnitude (TIRM) imaging in the midventricular basal inferior region (with maximal 54 ms relaxation time on T2-mapping), and focal late enhancement (**right**) (arrows) during the delayed phase.

**Figure 2:**
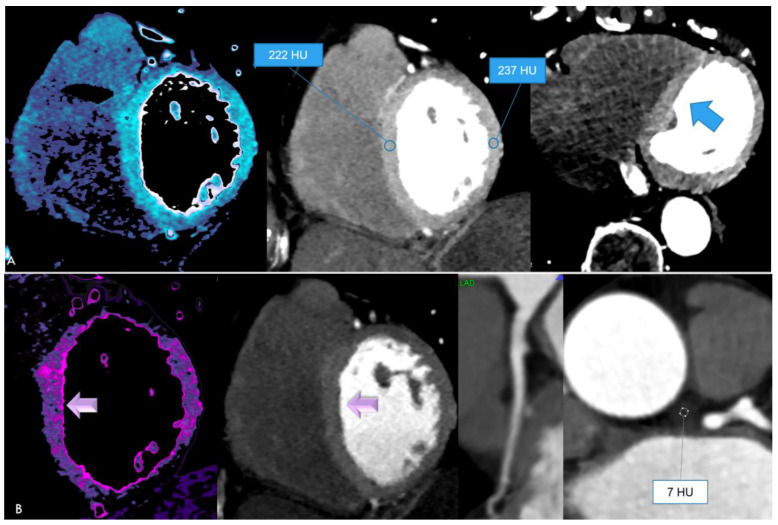
(**A**) Diffuse hyperenhancing myocardium (mean 222–237 HU) short-axis (**left**,**mid**) and axial view (**right**) with a small subendocardial hypoperfusion zone midventricular. (**B**) Short-axis view (**left**), normal left anterior descending (LAD) coronary artery (MPR) (**mid**), and an unusual pericoronary fluid collection (7 HU) surrounding the left main (LM) (**right**).

CTA findings in four acute care patients with signs of myocardial injury (troponin positive) were as follows:

Plaque rupture/possible spontaneous coronary artery dissection (SCAD)/MINOCA (n = 1) (Figure 1), hyperenhancing myocardium/MINOCA (n = 1) (Figure 2), and MINOCA (n = 3) [4]. All four (100%) patients had pericardial effusion. Three had mild pericardial effusion (<4 mm), a prominent superior epicardial recess “bat-sign” (>1 cm), and a positive PCAT. One had moderate pericardial effusion (>1 cm) and severe acute heart failure (LVEF 20%).

**Figure 3:**
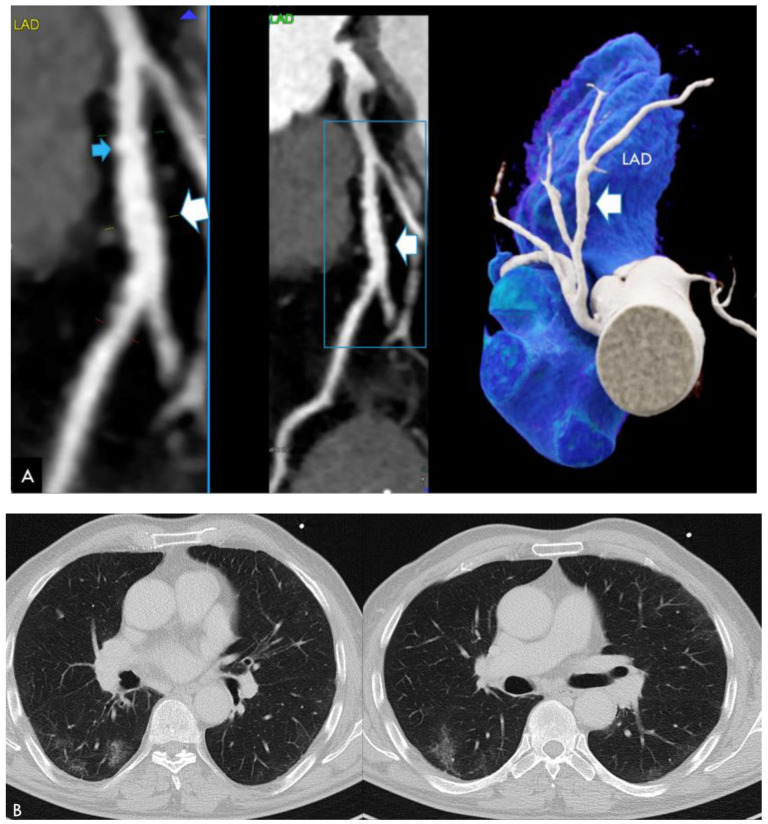
(**A**) Diffuse focal ectasia of the mid segment of the left anterior descending (LAD) (white arrow) with signs of atherosclerosis in a 71 year-old male active smoker with COVID-19 and mild lung involvement. Typical calcified plaque (blue arrow) of the LAD. (**B**) Patchy reticular ground-glass attenuation of the lower lung lobes with subpleural dominance, typical findings in COVID-19.

**Figure 4:**
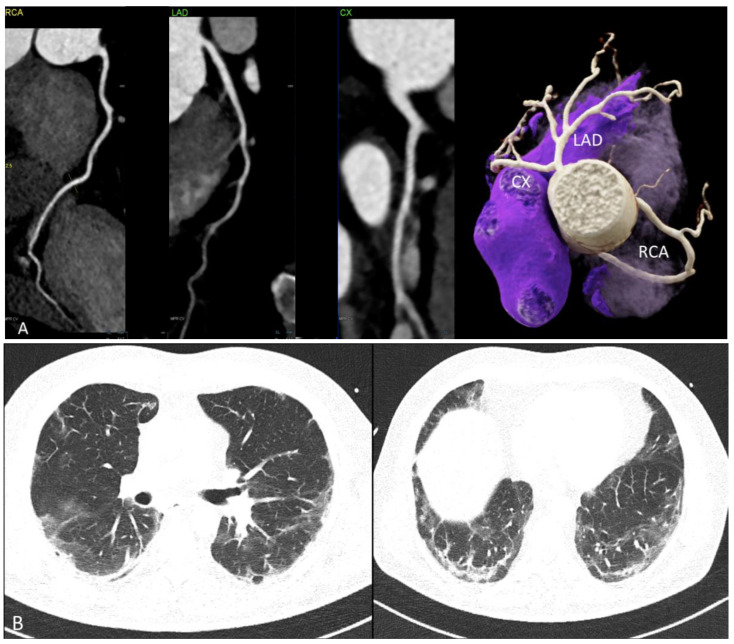
(**A**) Computed tomography angiography (CTA) showing normal coronary arteries (RCA, LAD and CX). (**left**: cMPR and **right***:* 3D-VRT). (**B**): Typical lung findings on chest CT: Persisting postinfectious lung injury (PILI) with reticulations and patchy ground glass opacification (GGO).

Among eight stable patients, mild pericardial effusion was found in only two (25%). PCAT was borderline positive in only two (25%). Two patients with high-risk plaque had lesion-specific positive PCAT. Typical atherosclerosis and obstructive coronary artery disease (stenosis > 50%) was ruled out in five (62.5%). Atypical vessel wall abnormalities suggestive for vasculitis were not observed (0%). A focal ectasia of the CX was found in one patient (12.5%) (Figure 3). Six of eleven (54.5%) patients had typical COVID-19 lung involvement (Figure 4).

Case 1 (Figure 1). A 36 year-old male with COVID-19 symptoms (39 °C fever) who tested positive for SARS-CoV-2 by real-time polymerase chain reaction (RT-PCR) developed chest pain at rest, with increasing severity on day 10. On admission, high-sensitivity (Hs)-troponin T was positive and peaked to a maximum 213 ng/dL. ECG showed transient non-consistent ST elevation during transfer to our hospital. Finally, non-STEMI was diagnosed.

Coronary CTA showed a non-calcified lesion in the mid right coronary artery (RCA) with 86 HU and 40% area stenosis. No other atherosclerotic lesions were present. Coronary artery calcium score was zero. PCAT was positive (−21 HU) for perivascular edema adjacent to the lesion, but negative along all other vessels. Coronary CTA imaging findings might resemble plaque rupture. A possible differential diagnosis would be spontaneous coronary artery dissection (SCAD) with subsequent wall thrombosis. Invasive coronary angiography, performed within 1 day, could not confirm SCAD. However, SCAD might have occurred already 10 days ago and the lesion could represent wall hematoma. Other CTA findings were mild pericardial effusion with a prominent superior pericardial fluid recess (>1 cm) (“bat-sign”). C-reactive protein (CRP) was normal. COVID-19 lung involvement was mild.

Cardiac magnetic resonance imaging (MRI) showed edema and late enhancement in the inferior myocardium, establishing the diagnosis of myocardial infarction with nonobstructive coronary arteries (MINOCA). The patient was treated with acetylsalicylic acid (ASA) 100 mg and discharged after 3 days.

Case 2 (Figure 2). A 76 year-old-female was referred to our hospital due to an episode of atrial fibrillation. RT-PCR was positive for SARS-CoV-2 10 days ago.

Hs-troponin was negative on admission, and increased mildly from 17.2 to peak 34.4 ng/dL (upper normal value: 14 ng/dL). Cardiac enzymes (CK-MB) remained negative. C-reactive protein (CRP) was mildly increased (1.43 mg/dL) (upper NV: 0.5 mg/dL).

Coronary CTA was performed due to rising Hs-troponin and intermittent chest pain, and arterial hypertension. Coronary arteries were normal, obstructive coronary artery disease was ruled out. An unusual, highly diffuse myocardial enhancement of 222–237 HU was the most remarkable CTA finding, with a subtle subendocardial hypoperfusion zone midventricular suggesting microvascular thrombosis. D-dimer was moderately elevated (721 ng/dL). There was an unusual pericoronary fluid collection surrounding the left main (LM) (+7 HU). PCAT was positive for edema surrounding the LM (mean, −21 HU), but not along all other vessels. She had mild COVID-19 pneumonia. She was treated with a novel oral anticoagulant (NOAK)(Apixaban, 5 mg) and discharged after 17 days.

CASE 3 (Figure 3). This case involved a focal ectasia of the left anterior descending (LAD) in a 74 year-old male who presented with stable chest pain 37 days after COVID-19. He was an excessive active smoker and had typical atherosclerosis (calcified plaque) (see Figure 3A). There was mild residual COVID-19 lung involvement (see Figure 3B). PCAT was positive (−51 HU) adjacent to the mid LAD, but not the RCA.

CASE 4. (Figure 4) A 58 year-old male with recurrent chest pain presented 51 days after a severe COVID-19 course including an intensive care unit (ICU) stay. The coronary vessels had normal walls, no irregularities, no focal or diffuse ectatic segments, and no signs of atherosclerosis. Obstructive CAD (stenosis > 50%) was ruled out (RCA = right coronary artery; LAD = left anterior descending; and CX = circumflex artery) (see Figure 4). PCAT was normal (−78 up to −116 HU) (Figure 4A).

Persisting postinfectious lung injury (PILI) with reticulations and patchy ground glass opacification (GGO) were found bilaterally, with the lower lobes having a typical appearance for COVID-19 ( Figure 4B).

**Figure 5:**
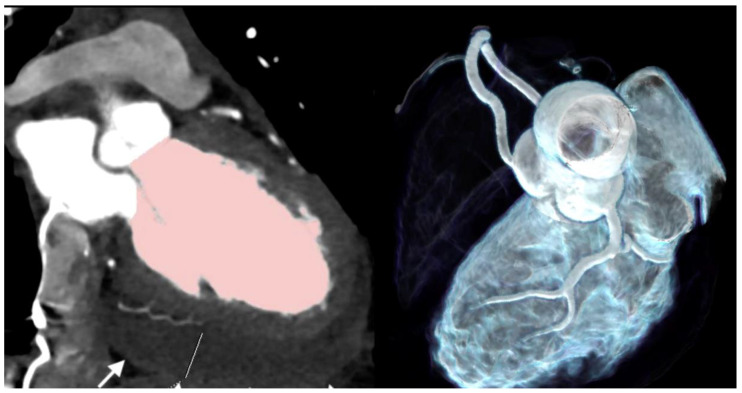
CT images (**left**), white arrow pointing at pericardial effusion (sagittal oblique plane), and an enlarged LV ((**right**), 3D VRT) with severe hypokinesis, an LV-EF of 20%, and normal coronary arteries (**left**).

CASE 5. (Figure 5) A 39 year-old female presented to our ED with severe heart failure, tachycardia, and low blood pressure on day 4 after her first positive RT-PCR for SARS-CoV-2 during Omicron B4/5 dominance in our geographic region. The prior clinical course was mild, with slight join pain, chest discomfort, and nausea. She had no fever on admission (35.5 Celsius) and no respiratory symptoms. C-reactive protein was normal and interleukin (IL)-6 was elevated. CK was elevated, Hs-troponin T (640 ng/dL), and brain natriuretic peptide (15.904 ng/dL) were elevated. The resting electrocardiogram (ECG) showed ST elevations; therefore, coronary CTA was performed to rule out an ischemic origin. Coronary CTA showed normal coronary arteries, moderate pericardial effusion (arrow), and a poorly contracting dilated left ventricle with a very low LV-EF of 20%. She was immediately transferred to the ICU. There was no typical COVID-19 pneumonia on the large field of view (FOV) chest CT reconstructions.

**Figure 6:**
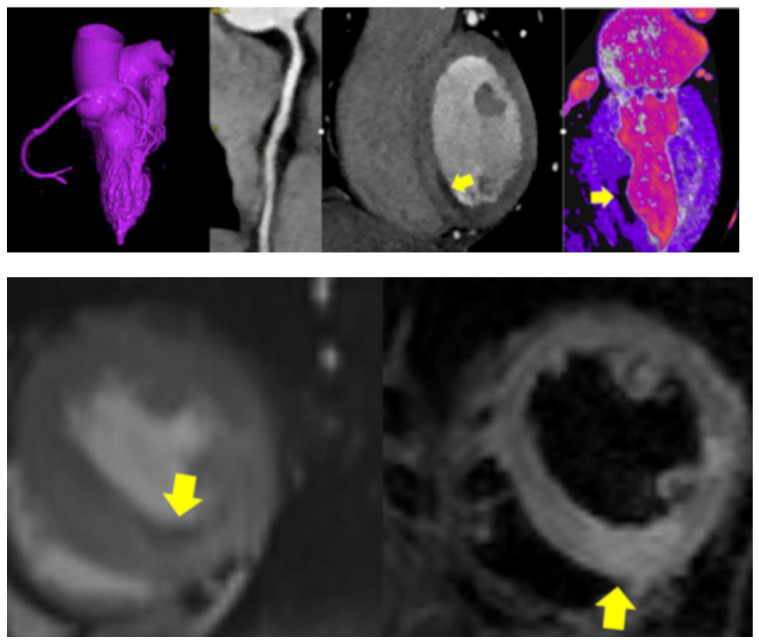
CTA showed diffuse vessel wall irregularities with hazy wall thickening of the RCA (**upper panel, left**), and signs of perivascular inflammation (PCAT −44 HU). Both CTA (**upper panel**) and MRI (**lower panel**) showed midventricular inferior perfusion defects (hypodense, yellow arrow) (**left**) and myocardial edema (**right**) inferior on T2 MRI sequences (yellow arrows). (**upper panel**): coronary computed tomography angiography (CTA) and (**lower panel**): magnetic resonance imaging (MRI) with perfusion sequence (**left**) and T2 (**right**). A final diagnosis of MINOCA was made.

CASE 6. (Figure 6) A 48 year-old female presented with SARS-CoV-2-infection and acute typical chest pain, elevated troponin, and negative T-waves on ECG (inferior leads) with a final diagnosis of NSTEMI-ACS.

## 4. Discussion

In acute care patients with signs of myocardial injury, there were three major CTA findings.

The first finding was plaque rupture with perivascular inflammation (Figure 1) and MINOCA. The CTA findings may resemble plaque rupture. The density of the lesion was rather high (86 HU). There was no lipid-necrotic “high-risk” low attenuation plaque component (<30 HU). A higher intralesional HU of a hypodense plaque may indicate intraplaque hemorrhage or thrombotic appositions. A possible differential diagnosis would be SCAD with intramural hematoma. However, SCAD was ruled out by invasive coronary angiography in our patient. In this patient, cardiac MRI showed edema and a late enhancement of the inferior myocardium (Figure 1), confirming acute myocardial necrosis related to the lesion territory. Perivascular edema was present on CTA, suggesting focal perivascular inflammation [3]. The patient was young, had no cardiovascular risk factors and no predisposing factors for SCAD. Whether COVID-19 infection has triggered plaque rupture or not remains speculative, but would be reasonable, because perivascular inflammation is regarded as a driving force for increasing plaque instability and the risk of plaque rupture [2,3]. In general, SCAD is more common in females, arterial hypertension, and fibromuscular dysplasia [9], therefore the lesion may instead represent plaque rupture during SARS-CoV-2 infection.

Another acute patient of our cohort also presented with MINOCA [4]. MINOCA due to microthrombi causing myocyte necrosis is well documented and the most common cardiac complication [1] of COVID-19. Cardiac magnetic resonance imaging (CMR) is the modality of choice for diagnosis. The utility of coronary CTA is to rule out obstructive coronary artery disease (CAD). However, importantly, coronary CTA should be performed only in the case of an urgent clinical suspicion (e.g., chest pain, myocardial injury, and cardiovascular risk factors). Invasive coronary angiography can be avoided, minimizing the risk for both patient and healthcare personnel exposure to SARS-CoV-2.

Second, diffuse hyperenhancing myocardium (Figure 2) was observed in one of our acute care patients. This was the most unusual finding, with remarkably high CT-attenuation values (237 HU). The patient had signs of myocardial injury and chest pain. A subtle subendocardial myocardial hypoperfusion zone midventricular was present, indicating microvascular dysfunction/thrombosis. D-dimer was elevated. The hyperenhancement might be explained by a combination of inflammation and vasodilatation during hyperinflammatory phases, triggered by cytokine storms during stage 3 of COVID-19 [10]. Such hyperenhancement is not typical for viral myocarditis, which causes patchy hypodense first-pass perfusion defects. Myocarditis in COVID-19 has been reported as having a diffuse, rather than a focal, pattern [11].

A slight variation in HU pending on kV settings must be considered, but >222 HU is certainly too high to be interpreted as normal even for low (80–100 kV) settings. There was also evidence of perivascular edema (PCAT < −70 HU). An unusual fluid collection surrounding the LM was found. Both most likely reflect signs of vasculitis, which were only found in acute patients, but not in those with stable chest pain and no myocardial injury.

Third, perimyocarditis [11,12,13] is another complication during COVID-19. Mild pericardial effusion with or without prominent superior epicardial recesses (“bat-sign“) (Figure 1C) was less common in stable patients, but consistently present in acute care patients. A total of 2.3% of competitive athletes presented with myocarditis on MRI between 10 and 77 days [13]. T2 elevation resolved in 100%, and late gadolinium enhancement (LGE) resolved in 40.7%. One patient had moderate pericardial effusion and severe heart failure (EF 20%) during the early acute phase of infection (Figure 5), with normal CRP. Therefore, coronary CTA was performed to rule out underlying coronary obstructions due to occult CAD or thromboemboli for differential diagnosis.

Focal ectatic coronary segments (Figure 3) are non-specific findings, which can develop during or after any disease causing perivascular inflammation and vasculitis (Kawasaki disease, multisystemic inflammatory syndrome (MIS) after COVID-19 [14,15], systemic vasculitis (IgG-4 mediated) or other autoimmune, rheumatic diseases, or psoriasis [16], or HIV infection [17]). In the context of atherosclerosis, perivascular inflammation occurs if chronic inflammation is present, such as in diabetics or smokers [18]. Only one case was found in our cohort, who was an excessive active smoker. Therefore, focal ectasia could also have been a result of heavy smoking in this patient.

Pericoronary fat attenuation (PCAT) is a novel marker for perivascular inflammation [3], which can be positive in both atherosclerosis (“high-risk” plaque) and other systemic disease causing vasculitis. PCAT was positive (<−70 HU) in acute patients only, but not in stable ones.

In summary, the main use of CTA in our case series was to rule out—or to detect underlying CAD—in symptomatic patients with a clinical suspicion of CAD during or after COVID-19 (Figure 4). Coronary CTA is the modality of choice for the non-invasive diagnosis of CAD [2,19], due to its high accuracy for excluding and detecting obstructive CAD (>50% stenosis). Coronary CTA is nowadays a Class I indication for the non-invasive evaluation of patients with low-to-intermediate pre-test probability of coronary artery disease (CAD) according to the European Society of Cardiology (ESC) guidelines from 2019 [2]. Coronary stenosis can be classified as minimal (<25%) (CAD-RADS 1), mild 25–49% (CAD-RADS 2), moderate (50–69%) (CADRADS 3), severe (70–99%) (CAD-RADS 4), and occluded (CAD-RADS 5) according to the CAD-RADS classification [20]. Beyond stenosis severity, coronary CTA offers the advantage of plaque quantification and characterization. Low attenuation plaques (LAP < 30 HU) indicate a lipid-rich core (“vulnerable lesion”) and are strong independent predictors for CV events [21,22,23,24]. Non-obstructive CAD with less than 50% stenosis is now also regarded as a novel important diagnosis, in order to define primary preventive measures [2,25]. Importantly, the presence of CAD, even in the absence of coronary artery calcium (Score 0), is strongly age dependent [26]. In patients less than 40 years, CAD is more common, even if calcium score is zero, than in older patients > 70 years. Further, of note, the presence of high-risk plaque features increases the likelihood of ischemia and chest pain symptoms even in patients with intermediate or non-severe stenosis [27]. CTA is also useful in patients with acute chest pain without troponin elevations and intermediate pre-test probability according to AHA guidelines [7], and is discussed as a useful alternative modality in selected patients presenting with NSTEMI-ACS in the diagnostic work-up, due to its ability to detect, or rule out, obstructive CAD [28]. While MRI is the reference modality of choice for the diagnosis of MINOCA, CTA was a useful modality as an alternative in patients with acute COVID-19 infection, troponin elevations, and suspected myocardial injury of unclear etiology, especially during the early pandemic phase with restricted healthcare resources during nationwide lockdowns [29].

In addition, acute and postinfectious lung injury [30] can be detected and characterized from coronary CTA by applying the additional “large field of view (FOV)” including lung window reconstructions. This is certainly relevant in post-COVID-19 patients presenting with persisting chest pain and dyspnea complaints.

Finally, novel technologies such as photon-counting CT enable multi-energetic spectral imaging [31,32] which provides further advancements for cardiovascular imaging. The quantification of the extracellular volume (ECV) by CT [33] allows for the differentiation of fibrosis and edema. Further, late enhancement CT improves myocardial tissue characterization by enabling the imaging of myocardial fibrosis and inflammation at a low radiation dose of <1 mSv by using high-pitch CT scanning [34], providing an alternative to MRI if a patient presents with contraindications. Further, photon-counting CT improves spatial resolution by applying an “ultra-high-resolution (UHR)” mode [31,32], and as a result, small coronary plaques can be better delineated, and artifacts from calcifications reduced and radiation exposure reduced [35] even for ECV imaging.

## 5. Conclusions

Acute CTA findings during COVID-19 infection were myocardial infarction with non-obstructive coronary artery disease (MINOCA), plaque rupture/SCAD, and hyperenhancing myocardium and perivascular edema suggestive of vasculitis; these indicate the need for further systematic research.

Abnormal fluid in the superior epicardial recesses and mild pericardial effusion were the most common findings in acute patients. Pericardial effusion was less common in stable patients, and there were no evident signs of vasculitis in stable patients.

## Figures and Tables

**Table 1:** Study cohort and CTA findings (n = 12 patients).

**Cohort Profile**		
Age (years)	54.8 ± 22	
Females	4 (33.3%)	
BMI (kg/cm^2^)	23.05 ± 3.35	
	(range, 17.6–27.5)	
Overweight > 24 kg/m^2^	4 (33.3%)	
Obese > 30 kg/m^2^	0 (0%)	
**Cardiovascular risk factors**		
Arterial Hypertension	4 (33.3%)	
Smoking	0 (0%)	
Positive family history	0 (0%)	
Dyslipidemia	1 (8.3%)	
Diabetes	2 (16.6%)	
**Comorbidities**		
Atrial fibrillation	1 (8.3%)	
COPD	1 (8.3%)	
**Acute congestive heart failure**	1 (8.3%)	
**Medication**		
Antihypertensive	4 (33.3%)	
Anticoagulation	1 (8.3%) NOAK	
	1 (8.3%) clopidogrel	
Statin	1 (8.3%)	
**Time to positive RT-PCR for SARS-CoV-2**	15.1 days (range, 0–51)	
**Clinical presentation**		
Acute, unstable patients	4 (33.3%)	
Stable patients	8 (66.6%)	
Hs-troponin max. in acute patients (n = 4)	1789 ng/dL 50 ng/dL 624 ng/dL 30 ng/dL	
**CTA findings**		
	Acute, unstable N = 4	Stable N = 8
Pericardial effusion	4 (100%)	2 (25%)
Plaque rupture/ACS	1 (25%)	0 (0%)
MINOCA	2 (50%)	0 (0%)
Hyperenhancing myocardium	1 (25%)	0 (0%)
Perivascular inflammation (PCAT+)	4 (100%)	2 (25%)
**Coronary artery disease by CTA**	1 (25%)	4 (50%)
Nonobstructive (<50% stenosis)	0 (0%)	2 (25%)
Obstructive (>50% stenosis)	1 (25%)	2 (25%)
Diffuse vessel wall irregularities	2 (50%)	3 (37.5%)
Focal ectasia	0 (0%)	(12.5%)

**Abbreviations**: MINOCA = myocardial infarction and non-obstructive coronary artery disease. PCAT = pericoronary fat attenuation. ACS = acute coronary syndrome. CTA = computed tomography angiography. COPD = chronic obstructive pulmonary disease. BMI = body mass index. Hs = high sensitivity. NOAK = novel oral anticoagulant.

## Data Availability

Data is contained within the article.
